# Conservation Units for Anadromous Arctic Char (*Salvelinus alpinus*) in the Canadian Arctic Informed by Genetic Structure, Population Connectivity and Adaptive Genomic Variation

**DOI:** 10.1111/eva.70259

**Published:** 2026-05-24

**Authors:** Xavier Dallaire, Anne Beemelmanns, Les Harris, Colin P. Gallagher, Ross Tallman, Jean‐Sébastien Moore

**Affiliations:** ^1^ Institut de Biologie Intégrative et Des Systèmes Université Laval Québec Canada; ^2^ Centre D'études Nordiques Université Laval Québec Canada; ^3^ Ressources Aquatiques Québec Université du Québec à Rimouski Québec Canada; ^4^ Fisheries and Oceans Canada Arctic Fisheries and Marine Mammal Science Division Winnipeg Manitoba Canada

## Abstract

Intraspecific genetic diversity is a crucial aspect of biodiversity conservation as it preserves evolutionary potential and enhances resilience to environmental change. Genomic‐informed delineation of Conservation Units (CUs) offers ways of subdividing species into groups based on demographic independence and adaptive differentiation, to develop biologically relevant conservation and management policies. CUs have been defined in many species of harvested anadromous salmonids, but broad‐scale data remain lacking in the Canadian Arctic, where anadromous Arctic Char ( *Salvelinus alpinus* ) dominates catches in Indigenous‐led subsistence and commercial fisheries. In this study, we use low‐coverage whole‐genome data from 30 Canadian Arctic Char populations to define CUs based on population structure, connectivity, and adaptive genetic variation. We highlight two main genetic groups, each of which comprises three subgroups, or candidate CUs: the North (above the 67th parallel), including the North Baffin Island, Kitikmeot, and Inuvialuit Settlement Region CUs; and the South (below the 67th parallel), including the South Baffin Island, Ungava Bay, and Hudson Bay CUs. This delimitation is supported by areas of low effective migration between candidate CUs, as well as isolation‐by‐environment, which suggests adaptive differentiation. Finally, we discuss opportunities and caveats related to genetic linkage when identifying adaptive variation from whole‐genome sequencing data using genome scans and gene–environment associations.

## 1 Introduction

Given the numerous threats to global biodiversity, there has been a call for conservation at the levels of ecosystems, species, and within‐species genetic diversity (Laikre et al. 2020). Of these three levels, species conservation has gathered much of the attention, while assuming a simplified categorization of nature and offering a common metric of biodiversity. In practice, the delimitation and definition of species is rarely straightforward (Mace 2004; Frankham et al. 2012; Garnett and Christidis 2017), and speciation proceeds via gradual genomic divergence among populations, often promoted by restricted gene flow and driven by the combined effects of genetic drift and natural selection in contrasted habitats (Roux et al. 2016). Both these evolutionary forces are subject to fluctuations over long timescales, as populations reconnect in secondary contact zones, or changes in the environment shift selective pressures (Seehausen et al. 2008; Rosenblum et al. 2012). The resulting intraspecific genetic diversity is a key component of biodiversity that warrants protection, as it underpins evolutionary potential and enhances resilience to environmental change and anthropogenic pressures (Forest et al. 2007; Schindler et al. 2010). Similar recognition of the conservation value of intraspecific diversity has emerged across taxa and regions, including marine fishes, terrestrial vertebrates, and plants, where genomic data regularly inform management decisions at sub‐species levels (Hoban et al. 2020; Allendorf 2025).

Biological systems are inherently complex, and speciation is best understood as a continuous process. Nevertheless, discrete intraspecific classifications remain necessary to inform conservation priorities within limited budgets and to guide the management of harvested species. There exists a vast body of literature on the delineation of Conservation Units (CUs), which generally relies on the identification of demographic independence (leading to genomic divergence) and adaptive differentiation (Waples 1991; Moritz 2002; Coates et al. 2018). Among the numerous CU models developed over the years, the Evolutionary Significant Unit (ESU; first proposed by Ryder 1986) aims to provide a framework for defining subspecific groups that represent both unique genetic variation and the historical or ongoing connectivity among populations within a species. Depending on its definition, it can either be based exclusively on genetic characters or incorporate ecological data (Fraser and Bernatchez 2001). ESUs can be further subdivided into Management Units (MU), defined as demographically independent populations identified based on contemporary gene flow and population dynamics rather than deep evolutionary history. This subdivision allows managers to implement local actions, such as sustainable harvest levels, supplementation, restoration, or monitoring, that can maintain genetic diversity, reduce inbreeding, buffer against environmental fluctuations, and support the long‐term viability of the broader ESU (Moritz 2002).

The concept of the ESU has been translated into legislation in multiple countries (e.g., the USA “Endangered Species Act” and the Australian “Endangered Species Protection Act”). In Canada, the “Species at Risk Act” applies a similar concept of Designatable Units (DU, Green 2005), which are considered discrete if there is evidence of limited transmission of heritable traits or markers between them, or if they are separated by a natural disjunction in their distribution. A discrete unit is then considered significant if its evolutionary trajectory dates back long enough to create an evolutionary history not found elsewhere in Canada (e.g., units originating from distinct glacial refugia) or if it possesses unique and verifiable adaptive traits (COSEWIC 2023).

Genetic data has been central to work related to CUs, as their delineation often revolves around a two‐step process: (1) describing the structure of populations, which is often hierarchical within species, and (2) choosing the level of distinctiveness appropriate for defining units, which implies some degree of subjectivity (Waples 1995). Cytoplasmic (both mitochondrial and chloroplastic) DNA has long been used to characterize intraspecific lineages (Avise et al. 1987), while microsatellites have contributed to investigating population structure and connectivity (Ellegren 2004). However, genomic data, that is, genome‐wide datasets produced by genotyping‐by‐sequencing (GBS, thousands of SNPs) or whole‐genome sequencing (WGS, millions of SNPs), have the potential to improve delineation of CUs by quantifying adaptive variation in addition to neutral variation (Funk et al. 2012). This is typically achieved scanning the genome for loci displaying higher differentiation than expected under neutral evolution, suggesting the imprint of natural selection, or by assessing correlations between allele frequencies and biologically relevant environmental variables (hereafter gene–environment associations, GEA, Rellstab et al. 2015; Dauphin et al. 2023).

Anadromous salmonids (i.e., those migrating from marine to freshwater habitats to spawn) represent a diverse group of fishes with significant economic and cultural importance worldwide. Their strong homing behavior (i.e., returning to natal sites for spawning, thereby limiting gene flow among populations) plays a crucial role in shaping their population structure and is a factor long recognized as key for their conservation and management (Thompson 1959). Many salmonid populations are facing strong harvesting pressures and are actively managed within CUs to ensure sustainability and long‐term population persistence. For instance, Atlantic Salmon ( *Salmo salar* ) along Canada's eastern coast are currently managed within 15 extant DUs (COSEWIC 2010; Moore, Bourret, et al. 2014; Moore, Loewen, et al. 2014). However, further subdivisions into 19 DUs were recently suggested based on genomic data (Lehnert et al. 2023). On the western coast, Pacific Salmons (*Oncorhynchus* spp.) are divided into dozens of CUs per species, with larger watersheds, such as the Fraser River in British Columbia, supporting multiple CUs (Xuereb et al. 2022; DFO 2024). Overall, CUs have proven essential for guiding effective fishery management and sustaining salmonid populations across diverse systems.

Northern Canada is home to populations of Arctic Char ( *Salvelinus alpinus* ), a salmonid whose anadromous form has long been the most harvested fish across Inuit Nunangat (i.e., the homeland of Inuit; Friesen 2004; Priest and Usher 2004). Arctic Char fisheries are predominantly small‐scale and harvested for subsistence purposes (Roux et al. 2019; Tallman et al. 2019), yet collectively amount to a high replacement value, i.e., the estimated cost of substituting the harvested fish with equivalent grocery‐bought protein (Brubacher 2004). While subsistence stocks are locally managed, some regions harbour commercial fisheries that are co‐managed between the Indigenous land claim body and the federal government (Fisheries and Oceans Canada). Arctic Char and sister species such as Dolly Varden ( *Salvelinus malma* ) have long posed a taxonomic challenge because of their exceptional phenotypic and ecological diversity, which has historically led to extensive splitting into nominal taxa (Johnson 1980; Klemetsen 2010; Taylor et al. 2025). For example, non‐anadromous populations in the British Isles were once described as up to 15 distinct subspecies based on morphological variation. While some of these taxa were later synonymized or reinterpreted as intraspecific forms within the Arctic Char complex (Adams and Maitland 2007; Barthelemy et al. 2023), others are still recognized in conservation frameworks such as the IUCN Red List (e.g., *Salvelinus youngeri* ). More recently, genomic approaches have delineated evolutionary significant units (ESUs) and management units (MUs) in these populations based on SNP data (Fenton et al. 2026), highlighting the complexity and ongoing revision of Arctic Char taxonomy.

North American genetic variation in Arctic Char has been investigated using mtDNA and microsatellites. These studies indicate that most anadromous North American Arctic Char populations carry only haplotypes from the Arctic mtDNA lineage, which might have survived in a High Arctic glacial refugium (Moore et al. 2015), with the Atlantic lineage also present in Labrador (alongside rarer haplotypes from the Acadian lineage; Salisbury et al. 2019) and Greenland (Jacobsen et al. 2022). However, recent whole‐genome data revealed that admixture in the nuclear genome between the Arctic and Atlantic lineage was a prominent feature of Canadian Arctic Char populations south of the 67th parallel, redefining the extent of the second contact between lineages (Dallaire et al. 2025). Other recent genomic datasets for the species were focused on spatial scales from local to regional and have been used to investigate questions relating to population structure and connectivity (Moore et al. 2017; Li et al. 2021), morphotype differentiation (Kess et al. 2021; Salisbury et al. 2023), and local adaptation (Madsen et al. 2019; Dallaire et al. 2021; Layton et al. 2021). Understanding how genomic structure and environmental variation jointly shape population differentiation in Arctic Char contributes not only to Canadian conservation planning but also to broader efforts to identify evolutionarily meaningful units across the species' circumpolar range.

In this study, we revisit the whole‐genome resequencing dataset introduced by Dallaire et al. (2025), including more than 1000 anadromous Arctic Char whole genomes in 30 sampling sites across the Canadian Arctic. While this previous work provided a detailed view of the phylogeography of the two main glacial lineages of Canadian anadromous Arctic Char, the present study focuses on patterns of population connectivity and adaptive genetic variation to define candidate CUs at a national scale. To inform these units, we specifically (1) characterize the hierarchical genetic structure of populations in relation to administrative regions in the Inuvialuit Settlement Region (ISR, Northwest Territories), Nunavut, and Nunavik (Québec), and (2) identify areas of lower gene flow. Then, we aim to (3) disentangle patterns of (i) isolation‐by‐distance, (ii) isolation‐by‐environment, and (iii) isolation‐by‐colonization (related to post‐glacial recolonization) that characterize this structure. Finally, we (4) detect signals of natural selection and explore patterns of population structure driven by putatively adaptive genetic variation using genome scans and Gene–Environment Associations. In this study, we delineate candidate Conservation Units as population groupings supported by concordant evidence from genome‐wide structure, reduced gene flow, and adaptive differentiation. We use the term Conservation Unit as an inclusive framework that integrates criteria derived from the ESU and DU concepts. These units represent empirically supported inferences intended to guide monitoring, stock assessment, and regional management decisions.

## 2 Methodology

### 2.1 Sequencing and SNP Calling

**FIGURE 1:**
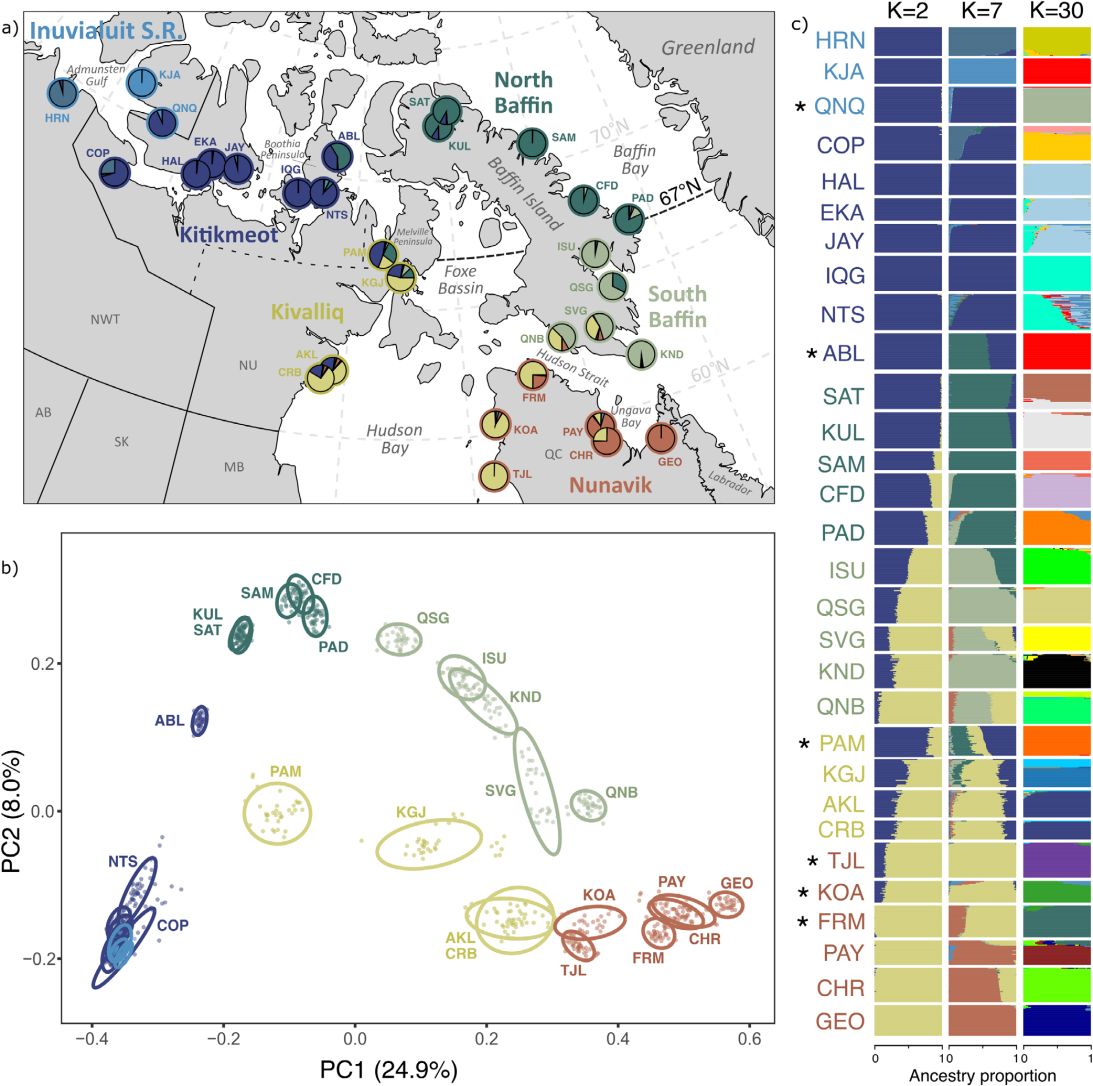
(a) Arctic Char sampling sites with colored labels showing their administrative region of origin. Pie charts show the site‐average ancestry proportions for *K* = 7 genetic clusters, as detailed in panel (c). (b) The first two axes of a principal component analysis for 277,570 independent SNPs. 95% confidence interval ellipses are drawn around each site, and the percentage of variance explained by each axis is noted in parentheses. (c) Individual proportion of ancestry for *K* = 2, 7, and 30 genetics clusters, CLUMPAK‐averaged over replicate runs in the major mode (out of 50 runs). Note that this figure reuses the same color palette for two codes: Administrative regions in panel (a) (labels, circles) and (b); and their most concordant genetic cluster (*K* = 7) in panel (a) (pie charts) and (c). An asterisk notes discordance between administration regions and genetic clusters. Additional colors for the *K* = 30 plot were randomly assigned.

We reanalysed sequencing data from 1016 Arctic Char from 30 Canadian populations (Table S1), published in Dallaire et al. (2025) (SRA project: PRJNA1031558). Briefly, whole‐genome sequences were obtained following Therkildsen and Palumbi (2017) on DNA extracted from fin clips from fish harvested at the mouth of rivers in the Inuvialuit Settlement Region (Northwest Territories), Nunavut, and Nunavik (Québec) between 2007 and 2023 (Figure 1a). As in Dallaire et al. (2025), data were processed following the pipeline described at https://github.com/enormandeau/wgs_sample_preparation: sequences were trimmed and aligned on the ASM291031v2 reference genome (likely from *Salvelinus malma* or a *S. alpinus* × *S. malma* hybrid; Christensen et al. 2021), duplicate reads were removed, indels were realigned and overlapping ends of paired reads were clipped.

Mapped and cleaned reads yielded an individual depth of coverage around 2X, and we estimated Genotype Likelihoods (GL) in ANGSD v0.937 (Korneliussen et al. 2014). GLs (or population allele frequencies derived from them) were used in all downstream analysis to avoid biases linked to strict genotyping using low‐coverage sequencing data (Lou et al. 2021). Analysed genomic positions were limited to those identified as polymorphic in Dallaire et al. (2025). Those positions were distributed on all assembled linkage groups, but SNPs in sex‐linked regions identified in Beemelmanns et al. (2025) were removed from all analyses. The SNP list was filtered for paralogs and other deviations from expected patterns due to transposable elements and ancestral autoploidization in salmonids, using *ngsparalog* (Linderoth 2018) following Dallaire et al. (2023). We kept SNPs with a global minor allele frequency above 1% and a minimum depth of coverage of 1X in at least 75% of individuals. We used *ngsLD* (Fox et al. 2019) to estimate linkage between SNPs within 500 kb, based on a random subset of half the samples. To create an LD‐pruned dataset, we then applied a graph‐based method to iteratively remove SNPs until no pairs within 200 kb exhibited an *r* ^2^ greater than 0.1.

### 2.2 Extraction of Environmental Data

We used the ArcGIS software v10.4 (ESRI 2011) to extract environmental data. Marine variables represent sea‐surface values for factors of potential biological importance for Arctic Char available in the BIO‐Oracle v3.0 dataset (Assis et al. 2024) and were averaged in a 20 km radius around each sampled river mouth and within 5 km from the coast. This standardized approach was used to characterize the local coastal environment in accordance with current knowledge of Arctic Char marine habitat use in Arctic Canada (Spares et al. 2015; Moore et al. 2016; Harris et al. 2020). We selected annual coastal averages for five marine variables (temperature, salinity, primary productivity, dissolved O_2_, and ice cover) as they represented good predictors for summer values, when Arctic Char occupies coastal habitats.

Freshwater variables were extracted from the RiverATLAS dataset by selecting the linear segment closest to the mouth of each sampled river, as these segments include values averaged over the entire upstream watershed. We initially considered seven variables: % of the watershed covered by lake or tree, average slope, sand fraction in the soil, and annual averages of discharge, air temperature, and precipitation. These variables are linked to both migratory challenges (e.g., slope, discharge, temperature) and spawning habitats (e.g., lake cover, sand in soil), two factors that may play an important role in local adaptation of Arctic Char (Moore et al. 2017; Dubos et al. 2023). Precipitation was later excluded because it was highly correlated with air temperature (Pearson *r* = 0.84), resulting in a final set of 11 environmental predictors (Tables S2 and S3, Figure S1).

### 2.3 Population Structure

When describing population structure in the dataset, we used a priori administrative regions to categorize sampling sites across the study area, namely the Inuvialuit Settlement Region (Northwest Territories); Kitikmeot, Kivalliq and Baffin (Nunavut); and Nunavik (Québec). We used the term Baffin instead of Qikiqtaaluk, as this administrative region also includes the High Arctic Archipelago, which was not sampled in this study. Note that anadromous populations of Arctic Char are less common at those latitudes where resident and landlocked Arctic Char dominate (Reist et al. 2013). North and South Baffin were further split around the 67th parallel. Note that the Pamiurluk Lake sampling site (PAM) was grouped within the Kivalliq region, despite technically being in Qikiqtaaluk, as this fish population is traditionally harvested by the people of Naujaat, a community in the Kivalliq region.

The overall distribution of genetic variation and population structure was initially evaluated through a principal component analysis (PCA) conducted on the LD‐pruned dataset using PCAngsd 1.10 (Meisner and Albrechtsen 2018). Subsequently, we examined the hierarchical population structure by applying NGSadmix (Skotte et al. 2013), estimating individual admixture proportions while varying the number of ancestral populations (*K*) from 1 to 30 (i.e., the number of sampling sites). For each value of *K*, we repeated the analysis over 50 independent runs and used the CLUMPAK algorithm (Kopelman et al. 2015) to group replicate runs that produced similar ancestry solutions (similarity score ≥ 0.85). For each K, we selected the group of replicate runs containing the largest number of runs (hereafter the major mode). Ancestry proportions were averaged across runs within this major mode and visualized using a stacked bar plot. To compare the fit of the model at different values of K, we (1) compared the proportion of runs in the major mode and (2) computed the *ΔK* (Evanno et al. 2005) by dividing the absolute value of the second‐order rate of change in the mean likelihood by the standard deviation of likelihoods for all runs of a given *K*.

Next, we used *ngsDist* (Vieira et al. 2016) on the unpruned dataset to compute genetic distances between every individual pair. We chose the uncorrected *p*‐distance, that is, the proportion of nucleotide sites (polymorphic in the dataset) at which they differ. SNPs with missing data in either individual of the pair were ignored. To express p‐distances as the proportion of genome‐wide differences between two individuals, we multiplied them by the ratio of the number of SNPs (4,044,588) to the total number of sites in the reference genome (1,516,033,356). We then built an unrooted neighbour‐joining tree on the distance matrix using the BIONJ algorithm (Gascuel 1997) in the ape v5.7 R package (Paradis and Schliep 2019). We visualized the tree with the R packages *ggtree* (Yu et al. 2017) and *ggtreeExtra* (Xu et al. 2021), while collapsing monophyletic groups of individuals from a single sampling site, then aligned individual ancestry proportions estimated with *ngsAdmix* for *K* = 2, 7, and 30 genetic clusters with the corresponding tree tips. These values of *K* were chosen as they represented Arctic vs. Atlantic ancestry as detailed in Dallaire et al. (2025) (*K* = 2), roughly corresponded to a priori geographic regions (*K* = 7), or matched the number of sampling sites (*K* = 30).

### 2.4 Connectivity, Isolation‐By‐Distance, Isolation‐By‐Environment

Dallaire et al. (2025) revealed strong patterns of isolation‐by‐distance that were distinct in groups of populations above (hereafter northern) and below (southern) the 67th parallel, with southern population pairs being more genetically distant at equal marine distances. Here, we revisited the relationship between genetic distance, as measured by the pairwise fixation index (F_ST_) and allele frequency difference (AFD), and either marine, environmental, and ancestry distance to investigate the interplay between (i) isolation‐by‐distance (IBD), (ii) isolation‐by‐environment (IBE), and (iii) isolation‐by‐colonization (IBC; defined as the proportion of genetic distance explained by the difference in genetic background contributions from two glacial lineages that recolonized the Canadian Arctic), respectively.

F_ST_, AFD, and marine distances were estimated as in Dallaire et al. (2025). Environmental distance between sites was calculated by performing a PCA on all environmental variables and measuring the Euclidian distance between population coordinates on the first seven axes, which encompassed 92.2% of the variation. Ancestry distance was measured as the difference in the average ancestry proportion for a sampling site from the NGSadmix analysis (*K* = 2) presented in Dallaire et al. (2025), which included Greenlandic populations and a Swedish outgroup descended from the Atlantic lineage. Said ancestry proportions ranged from 0 (pure Arctic lineage) in populations west of ABL to 0.77 (Arctic‐Atlantic admixed genetic background) in GEO and was used as a proxy of the proportion of ancestry from the Arctic and Atlantic lineages in the Canadian populations studied here.

The correlations between each measure of genetic distance and either marine, environmental, or ancestry distances was evaluated separately using simple Mantel tests as implemented in the R package *vegan* (Oksanen et al. 2022). We then used partial Mantel tests to assess whether each correlation remained robust when controlling for either type of distance (e.g., AFD ~ marine distance while controlling for ancestry distance, then for environmental distance, etc.). To control for the effect of two distance matrices conjointly, we first built a linear model between the genetic distance and admixture model while accounting for the population pair category (i.e., two northern populations, two southern populations, or one northern and one southern population). We then used the residuals as a substitute for genetic distance in a partial Mantel test with marine or environmental distance. To further investigate which component of the environment could contribute to isolation‐by‐environment, we repeated partial Mantel tests using the difference in the value for each environmental variable as environmental distance. We compared Mantel *r* statistics and their associated *p* values estimated from 999 permutations for tests including all population pairs, then only northern or southern pairs.

To characterize geographic areas of limited connectivity, we conducted an Estimated Effective Migration Surfaces (EEMS) analysis (Petkova et al. 2016) using FEEMS (Marcus et al. 2021). Briefly, the effective migration rate is comparable to the concept of effective population size (N_e_), in that it represents the migration rate at which an idealized stepping‐stone model at equilibrium would produce genetic dissimilarities equivalent to values observed in the data. In FEEMS, we modified the Python scripts *spatial_graph* and *run_cv* to skip the allele frequency estimation from genotypes and used the population MAF estimated in ANGSD as input since we did not call individual genotypes. We used the R package *dggridR* (Barnes and Sahr 2024) to create a triangular grid at resolution 7 (~55 km between cells) over the study area. To select the value for the smoothing parameter λ, we followed the leave‐one‐out cross‐validation method suggested in Marcus et al. (2021): we repeated the EEMS analysis after removing one population at a time with 20 values of λ varying from 0.1 to 10,000 and compared the migration surfaces for λ displaying local minima in cross‐validation errors.

### 2.5 Loci Under Putative Selection and Gene–Environment Association

SNPs with a global minor allele frequency over 0.05 were investigated for signatures of selection using *pcadapt* (Luu et al. 2017) and the XtX statistic in *Baypass v2.1* (Gautier 2015), and for Gene–Environment Associations (GEA) using the Bayes Factor statistic in *Baypass* and redundancy analyses (RDA).

We applied the *pcadapt* procedure in PCAngsd, which allows for individual genotype likelihoods as input. Briefly, *pcadapt* scans the genome using significant components of a PCA, here the first 15 PCs, as selected by PCAngsd. The multi‐dimensional z‐scores in output were converted to Mahalanobis distances using the orthogonalized Gnanadesikan–Kettenring method, corrected for genomic inflation following recommendations (Luu et al. 2017), then converted into *p* values via a χ^2^ test with 14 degrees of freedom. To build the input for *Baypass*, we converted allele frequencies estimated by population in ANGSD (−domaf 1) to allele counts by multiplying (then rounding) the frequencies of the major and minor allele by the SNP‐specific number of individuals (as given in the .mafs output from ANGSD, i.e., the number samples which passed the 1X coverage threshold at the SNP position) to jointly account for sampling size and missing data. We first sampled 10,000 random SNPs to compute a covariance matrix with the core model in *Baypass*, then used this matrix in the standard covariate model with every SNP (maf > 0.05) and all 11 environmental variables. We ran five replicate analyses and used the median XtX as an index of selection and the median Bayes Factor (BF) as a measure of the association between the allele frequency of a SNP and each environmental variable.

As a second method to detect gene–environment associations, we used redundancy analyses, which are constrained ordinations that assess the relationship between a multivariate response variable (here allele frequencies in sampling sites) and explanatory variables (the environmental factors). To account for collinearity among variables, we ran an initial RDA with all variables in the R package *vegan*, then iteratively removed the variable with the highest Variance Inflation Factor (VIF) until all VIF were under 10 (we only removed Dissolved O_2_ this way). We then ran backward selection on the model using the *ordistep* function, iteratively pruning variables until all retained predictors contributed to the model according to an ANOVA‐like permutation test (*p* < 0.1). Finally, we assessed the significance of RDA axes through an ANOVA‐like permutation test on the selected model and kept axes with *p* < 0.05.

The ancestry proportion from the Arctic and Atlantic lineages was a major determinant of the genetic variation in our study area (Dallaire et al. 2025) and strongly correlated with some of the environmental variables. As such, we explored the proportion of variation in MAF explained exclusively and jointly by (1) the 10 unrelated environmental variables, (2) the Arctic‐Atlantic ancestry proportion, as estimated by the NGSadmix analysis (*K* = 2) in Dallaire et al. (2025), and (3) geography, summarized by longitude and latitude of the sampling site. A variance partitioning analysis was performed using the varpart function in *vegan*.

We then conducted a partial RDA (pRDA) using the average ancestry proportion as the covariate. This covariate was chosen in the hope of avoiding overinterpreting shifts of allelic frequency that coincide with both the Arctic‐to‐Atlantic ancestry gradient and any correlated environmental gradient. On the other hand, using geography as a covariate has been suggested to lead to higher rates of false negatives when the environment is spatially autocorrelated (Günther and Coop 2013; Lotterhos and Whitlock 2015; Yeaman 2015). For the pRDA, we followed the same variable and axes selection steps as described above.

Every selection scan and GEA methods described above produced summary statistics by SNP that were aggregated by gene using a windowed Z analysis (WZA; Booker et al. 2024). WZA combines the information of multiple SNPs linked in a genomic region to assess if this region is in GEA or displays signs of selection. Since linkage varies across the genome, and in the absence of a robust recombination map, we opted to use genes annotated on the reference genome instead of windows of an arbitrarily defined length. We ran separate WZAs by using as summary statistics (i) the *p* values from pcadapt, (ii) the XtX and (iii) BF (for each environmental variable) from Baypass, and (iv) the squared loading on each significant axis of the RDA and pRDA. Briefly, those statistics were ranked, ordered and converted to empirical *p* values, then to *z*‐scores that were combined for SNPs inside each gene (i.e., between the start and end position based on the annotated reference genome) to compute a *p* value corrected for the number of SNPs in the gene (Booker et al. 2024).

For each WZA, we listed the top candidate genes (i.e., genes with *p* values < 0.001) showing the strongest signals of selection or association with the environment. To explore the relationship between our top candidate genes and regions of low recombination as inferred by local PCA in Dallaire et al. (2025), we conducted a series of χ^2^ tests to check if top candidates identified by each analysis were more likely than by chance to be found in long putative local ancestry tracts, then in other local PCA outlier regions not correlated with the admixture gradient between the Arctic and Atlantic (as listed in Table S4).

For each selection scan or GEA analysis, we repeated a PCA on genetic variation for LD‐pruned SNPs located inside top candidate genes. We then performed a gene ontology (GO) enrichment test with goatools 1.2.3 (Klopfenstein et al. 2018) on all top candidate genes, and on genes identified as top candidates by more than one method. We used a custom annotation table, obtained by mapping Arctic Char transcripts (accession GCF_002910315.2; ASM291031v2) against the Swissprot 1.2 database (UniProt Consortium 2024) with blast 2.12.0 (Camacho et al. 2009), according to the pipeline GAWN v0.3.5 (https://github.com/enormandeau/gawn). As we lacked the power to detect selection in WZA for genes with few SNPs, only genes for which we considered at least 5 SNPs were kept as the background set. Terms for biological processes (BP) with corrected *p* 0py7values (Benjamini and Hochberg 1995) under 0.05 were considered significantly enriched. Finally, we inspected a list of known quantitative trait loci (QTL) in salmonids, curated and aligned to the *S. Salvelinus* reference genome by Fenton et al. (2024), and listed those that fell within 100 kb of top candidate genes.

## 3 Results

### 3.1 Genetic Population Structure

Out of the 5.98 million polymorphic SNPs in Canadian and Greenlandic Arctic Char populations identified in Dallaire et al. (2025), 4.04 million passed the coverage filters and displayed MAF above 0.01 in Canadian populations alone. Subsequent LD‐pruning reduced this number to 277,570 SNPs. To facilitate knowledge transfer to local policymakers, populations were first categorized a priori by administrative regions of Inuit Nunangat: the Inuvialuit Settlement Region (ISR, Northwest Territories); Nunavik (Québec); and Kitikmeot, Kivalliq, and Baffin (Nunavut) (Figure 1a, circles and labels).

A principal component analysis revealed a population structure consistent with geography on the first two axes (comprising 32.9% of the variation; Figure 1b). However, most sites in ISR and Kitikmeot were densely clustered on these axes and while HRN and KJA were distinct on the fourth axis (3.6%), the rest of north‐western sites remained indistinguishable near the middle of the plot for the first 10 axes (totaling 55.8% of the variation; Figure S2). A neighbour‐joining tree based on *p*‐distances displayed a similar geographic pattern, with north‐western individuals on shorter branches than south‐eastern individuals (Figures S3 and S4). Many sampling sites clustered into exclusive groups, with the notable exception of closely located pairs of sites: EKA and HAL (Kitikmeot; 39 km, F_ST_ = 0.010, Table S5), AKL and CRB (Kivalliq; 64 km, F_ST_ = 0.011), and SAT and KUL (North Baffin; 65 km, F_ST_ = 0.014). Interestingly, PAY and CHR were only marginally further apart (69 km), but exhibited stronger structure (F_ST_ = 0.078). Finally, some sampling sites displayed considerable substructure, with their clade split into subgroups (e.g., SVG, KUL‐SAT) or a few individuals grouping into smaller, distant clades (e.g., NTS, KGJ).

**FIGURE 2:**
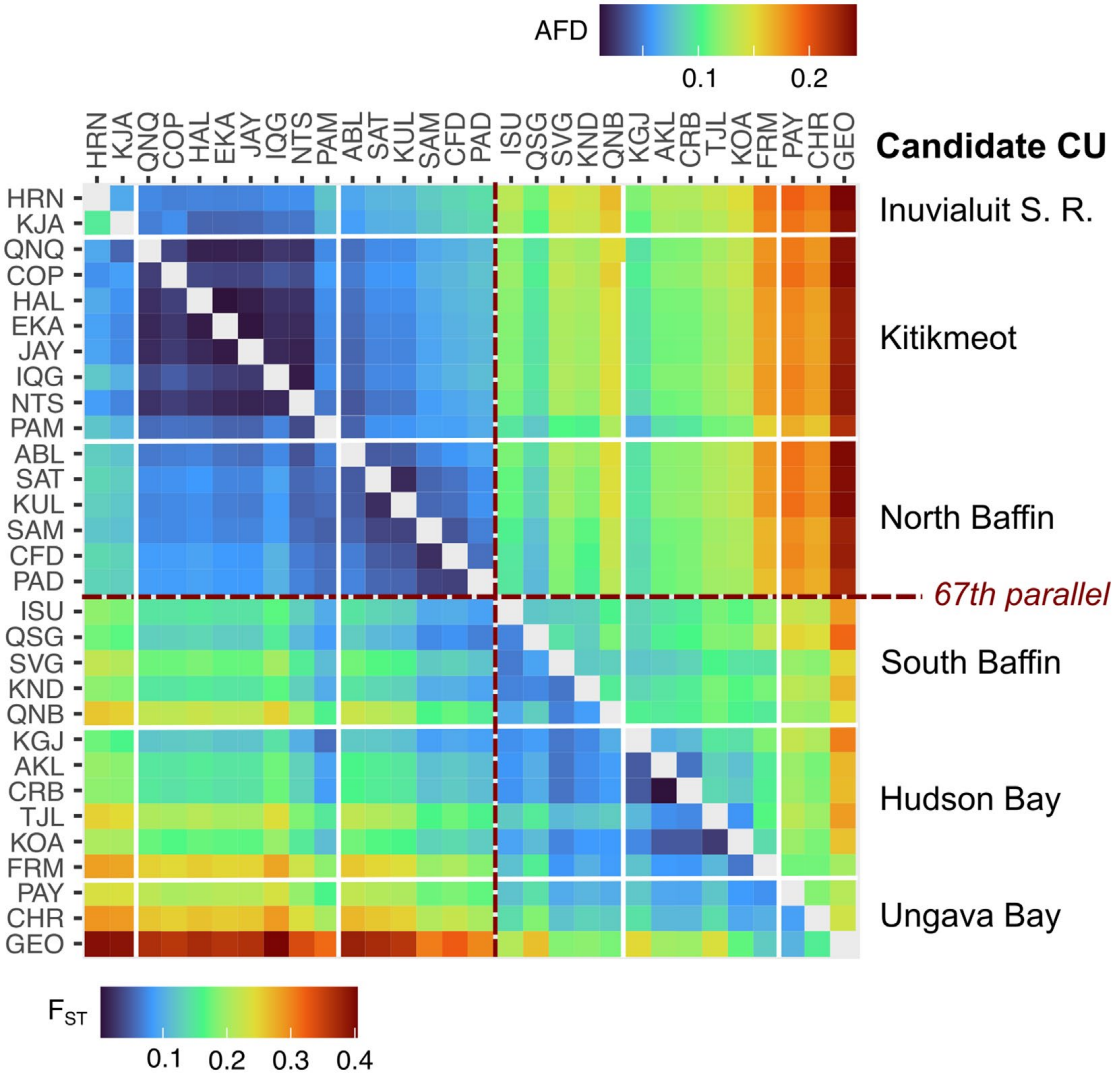
Allele frequency difference (AFD, above the diagonal) and F_ST_ (below the diagonal) between pairs of sampling sites. Candidate conservation units (named on the right) are divided by white solid lines, and the sampling sites above and below the 67th latitude line are divided by dark red dashed lines.

The *NGSadmix* analysis of ancestry proportions with *K* ranging from 2 to 30 ancestral populations, revealed a highly hierarchical structure (Figure 1c; Figures S5 and S6). At *K* = 2, eastern Nunavik and northern sites displayed pure ancestry for different groups, and sites in between followed a cline in admixture closely matching the pattern attributed to Arctic versus Atlantic lineage ancestry in Dallaire et al. (2025). At *K* = 30, the analysis discriminated most sampling sites, in line with clades identified in the neighbour‐joining tree. Interestingly, *K* = 7 groups produced a structure parallel to geographic regions defined a priori (Figure 1a, pie charts), with a few key discordances. For example, PAM clustered more closely with Kitikmeot than with Kivalliq populations, and ABL aligned with North Baffin rather than with Kitikmeot. Most notably, at this level of structure, populations along Hudson Bay and Ungava Bay clustered into different genetic groups, rather than aligning with the Kivalliq and Nunavik regions defined a priori. We hereafter refer to groups of sampling sites sharing a dominant genetic cluster at *K* = 7 as our proposed CUs. In contrast, NGSadmix runs for *K* = 6 showed inconsistent assignment for HRN and KJA, presenting either or both as a distinct group (Figure S6). As *K* = 7 resolved these populations into two distinct clusters, we grouped KJA and HRN within the Inuvialuit Settlement Region unit to avoid single‐population units, resulting in a total of six CUs (Figure 2).

### 3.2 Connectivity and Isolation

**FIGURE 3:**
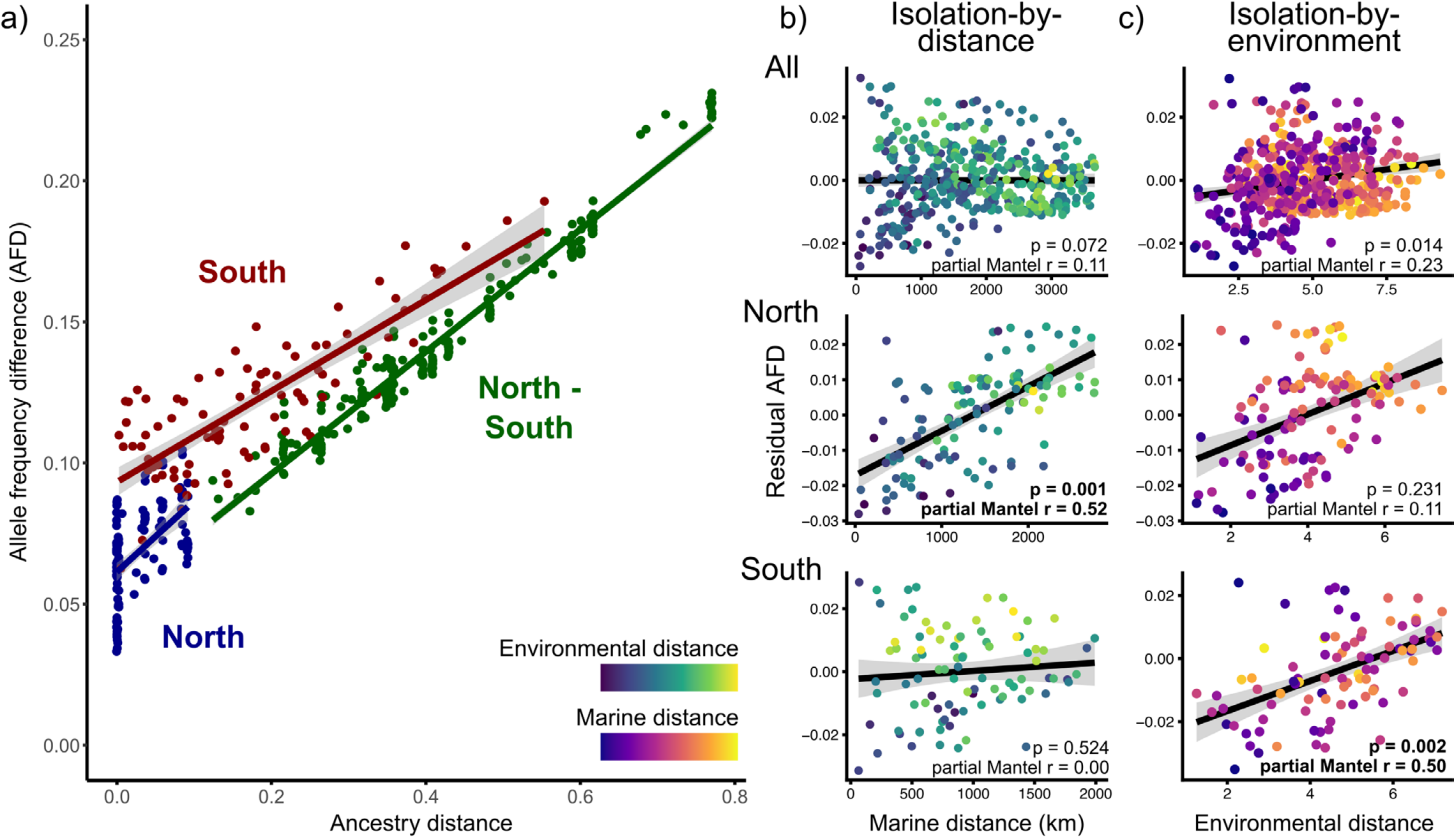
(a) Linear regression of allele frequency differences (AFD) between a pair of populations and their ancestry distance (i.e., the difference in their average ancestry proportion, NGSadmix, *K* = 2). Pairs were categorized depending on if they include two northern (blue), two southern (red), or one northern and one southern population (green), based on their position above or below the 67th latitude line. Partial Mantel tests were conducted between the residuals of the model from panel (a), and either (b) marine or (c) environmental distance for all populations (top), or either northern (middle) or southern ones (below). The remaining distance, that is, (b) environmental or (c) marine, was used as covariable and is displayed in a color gradient (lower values for the covariable, blue; higher values, yellow). Mantel partial *r* statistics are noted in bold when the associated *p* value was under 0.01.

Pairwise F_ST_ ranged from 0.011 (HAL‐EKA) to 0.407 (IQG‐GEO), while average allele frequency difference (AFD) in SNPs with MAF > 0.01 ranged from 0.033 (HAL‐EKA) to 0.231 (HRN‐GEO) (Figure 2; Table S5). The strongest predictor of genetic distance was lineage ancestry difference, with Mantel correlations (*r*) of 0.93 for F_ST_ and 0.90 for AFD (Figure 3a, Table S6). In comparison, environmental distance (summarized by a PCA of 13 factors) distance showed lower correlations (*r* = 0.59 for F_ST_, 0.61 for AFD), and geographical marine distances had even weaker, but still significant (*p* < 0.01), correlations (*r* = 0.48 for F_ST_, 0.54 for AFD) (Table S6). At equal ancestry, marine, or environmental distances, pairs of populations from the southern group were more genetically distant than northern pairs (Figure 3).

Partial Mantel tests indicated no signs of isolation‐by‐distance in the global dataset when correcting for both ancestry and environmental distance (Figure 3b, *r* = −0.02, *p* = 0.972), but signs of weak isolation‐by‐environment when correcting for ancestry and marine distance (Figure 3c, partial Mantel *r* = 0.23, *p* = 0.014). When repeating these analyses exclusively for northern or southern pairs, we revealed apparent isolation‐by‐distance in the North (Figure 3b, partial Mantel *r* = 0.52, *p* < 0.001), and isolation‐by‐environment in the South (Figure 3c, partial Mantel *r* = 0.50, *p* = 0.002). Despite a strong relationship between environmental and genetic distances in northern pairs, correcting for marine distances in a partial Mantel test decreased support for isolation‐by‐environment in the North (Figure 3c, partial Mantel *r* = 0.11, *p* = 0.23). Partial Mantel tests with specific environmental variables indicated that differences in slope (partial Mantel *r* = 0.33) explained a significant portion (*p* < 0.01) of the variation in study‐wide AFD. In contrast, differences in salinity (*r* = 0.43) explained a significant portion of the variation in AFD in southern pairs of sites (Table S6, Figure S7).

**FIGURE 4:**
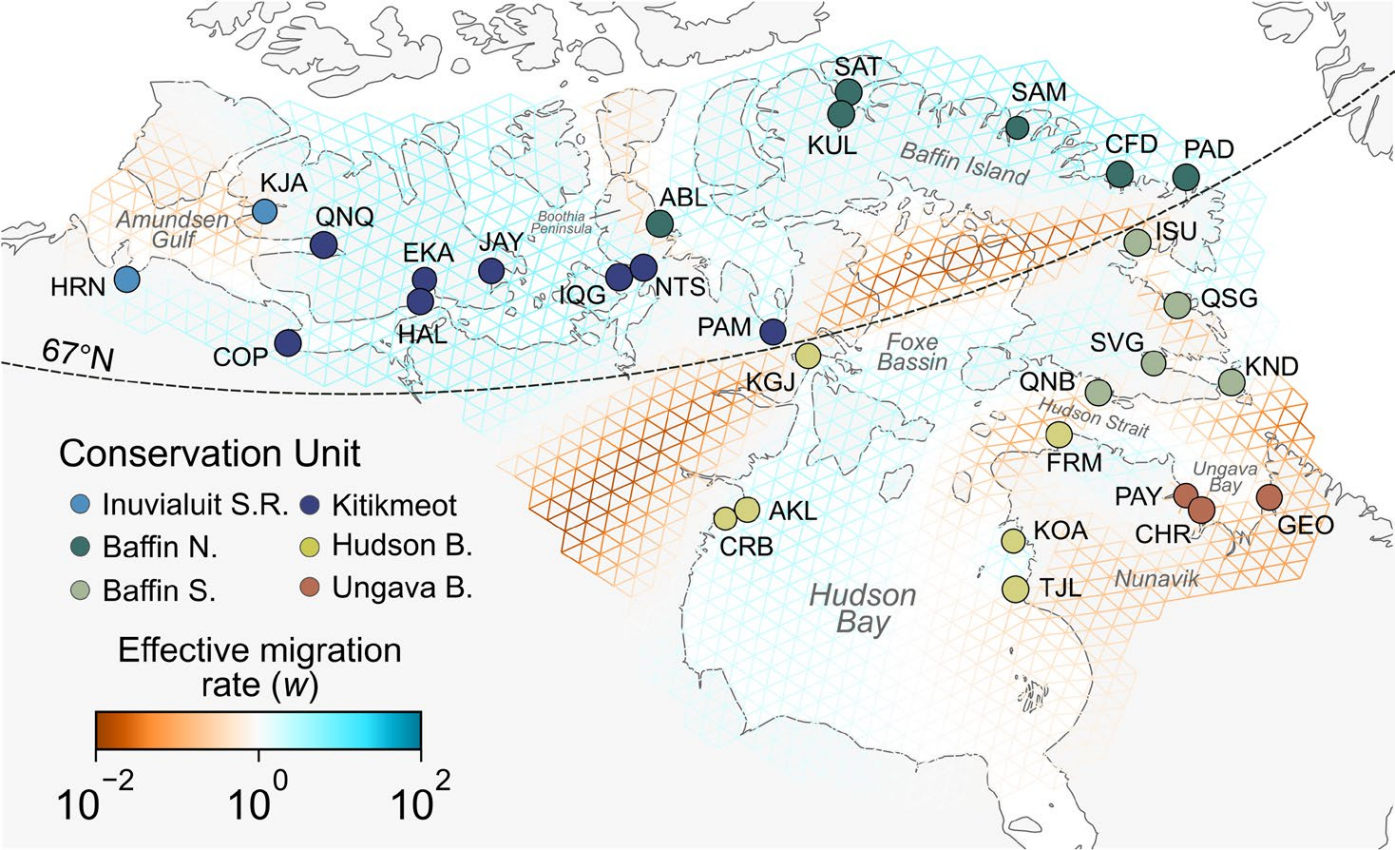
Edge‐specific effective migration rates (w) estimated from sampling allele frequencies in FEEMS with smoothing parameter λ = 21.5. Sampling sites were repositioned to the nearest node on a triangular grid (cell spacing ≈ 55 km) and colored with the proposed conservation unit (CU), following the most dominant genetic cluster in an NGSadmix analysis (*K* = 7), except for Inuvialuit S. R. (see Discussion for details).

We estimated effective migration rates across the study area from allele frequencies. The resulting migration map at different values of the smoothing parameter lambda mainly highlights an area of low effective migration between the previously described northern and southern regions (Figure 4; Figure S8). Smaller areas of low effective migration are observed over the Boothia Peninsula (between NTS and ABL), Amundsen Gulf (between HRN and KJA), Hudson Strait (between QNB and FRM), and inland Nunavik (between Hudson Bay and Ungava Bay). Sampling sites within CUs were generally connected by areas with effective migration rates exceeding 1.

### 3.3 Selection Scan and Gene–Environment Association

We investigated 2.6 million SNPs with MAF > 0.05 for signatures of selection, via pcadapt and the XtX statistic in Baypass (details below), or their association with environmental variables, with redundancy analyses without (RDA) and with (pRDA) correction for lineage ancestry, and the Bayes Factor statistic in Baypass.

**FIGURE 5:**
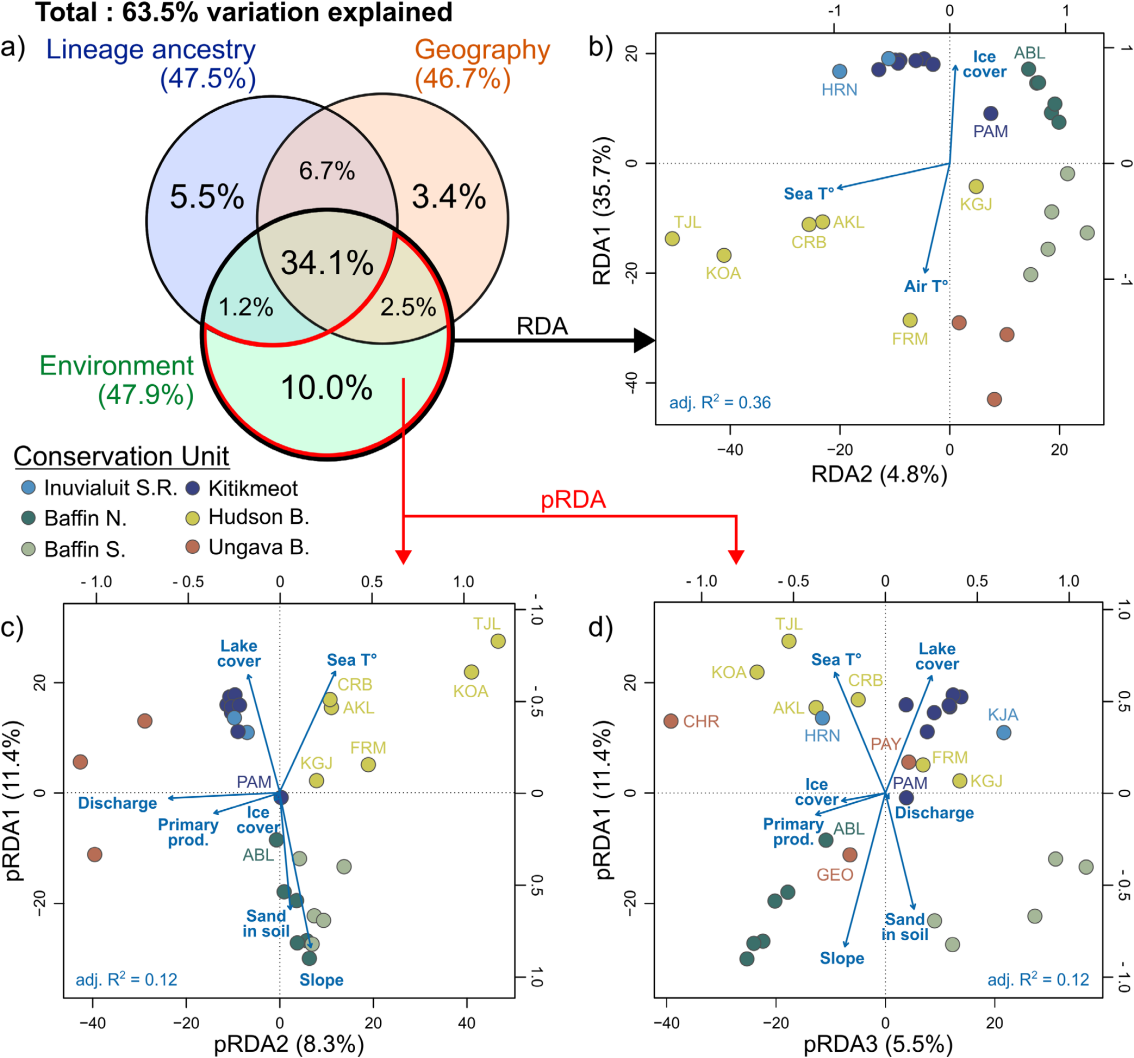
(a) Proportion of the variation in allele frequency explained independently and conjointly by a set of 11 environmental variables, glacial lineage ancestry (estimated by NGSadmix, *K* = 2), and geography (latitude and longitude). Biplots of (b) a redundancy analysis (RDA, 2 significant axes) and (c, d) a partial RDA accounting for the variation in ancestry (pRDA, 3 significant axes). Sampling sites are represented as points color‐coded by their proposed conservation unit, and vectors represent contributing environmental predictors (retained after model selection) according to the scales on the top and right axes.

A global RDA with all 11 selected environmental factors, Arctic‐Atlantic lineage ancestry (as estimated by the average ancestry proportion inferred with NGSadmix at *K* = 2), and geography (latitude and longitude) explained 63.5% of the variation (adjusted *R* ^2^) in MAF between sampling sites (Figure 5a). Environmental factors explained 47.9% of the variation, but a large part (37.8%) was conjointly explained by either lineage ancestry, geography, or both.

After backward variable selection with *ordistep*, the first two axes of the RDA (adjusted *R* ^2^ = 0.36) were significant (*p* < 0.05) and were largely correlated with (1) air temperature and ice cover; and (2) sea surface temperature (Figure 5b). The first three axes of the pRDA (adjusted *R* ^2^ = 0.12) were significant and were correlated with (1) sea‐surface temperature, lake cover, fraction of sand in soil, and slope; (2) discharge and primary productivity; and (3) primary productivity and ice cover (Figure 5c,d). RDA1 was highly correlated with latitude and lineage ancestry, whereas RDA2 and pRDA1 discriminated Baffin Island sites from the rest of the study area. Both RDA2 and pRDA2 also highlighted the genetic and environmental distinction between western (Hudson Bay: TJL, KOA) and Eastern (Ungava Bay: PAY, CHR, GEO) Nunavik.

**FIGURE 6:**
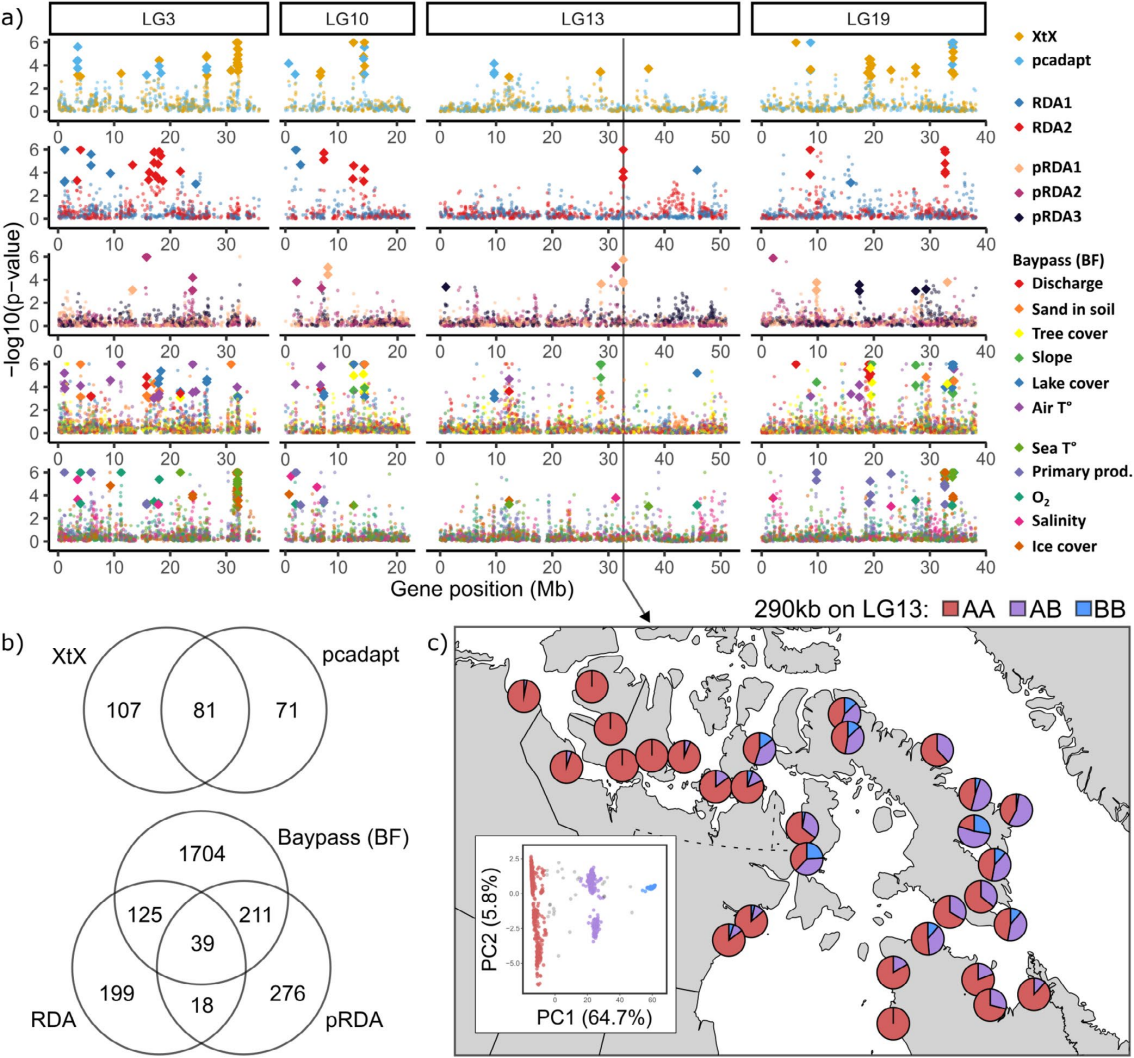
(a) Support for selection scans or gene–environment associations on genes with at least 10 SNPs on 4 linkage groups, in 5 panels regrouping methods: (1) XtX (Baypass) or pcadapt; (2) redundancy analysis axes (RDA); (3) partial RDA axes; and Bayes Factors (BF, Baypass) for (4) freshwater and (5) marine variables. Support at the gene level (empirical *p* values) was derived from combined per‐SNP summary statistics in a Windowed *Z* Analysis (WZA). Top candidate genes (*p* < 0.001) for more than one type of test were highlighted with larger diamonds than the other genes (small dots). (b) Venn diagrams presenting the number of top candidate genes (*p* < 0.001) for selection (top) or gene environment association (bottom) and the overlap in genes between methods. (c) Karyotype frequencies per sampling site for a 290 kb‐long putative inversion on LG13, as inferred by local PCA in Dallaire et al. (2025). The genomic position of the putative inversion is noted by an arrow in panel (a).

With a windowed Z analysis (WZA), we combined SNP‐specific statistics from the 5 methods (a total of 18 tests: pcadapt, XtX, 2 RDA axes, 3 pRDA axes, and 11 univariate tests for the BF statistics in Baypass) for 21,379 genes (with at least 5 SNPs) to provide rank‐ordered support for selection or GEA, in the form of empirical *p* values (Figure 6a). The genome scan methods (pcadapt and XtX) identified respectively 151 and 188 top candidate genes (*p* < 0.001), with significant overlap (81% or 31.2% of genes in common, Figure 6b). The GEA methods (BF, RDA, and pRDA) identified 2079, 381, and 544 top genes. BF showed notable overlap with the other methods: 164 (7.1%) of top genes were in common with the RDA and 250 (10.5%) with the pRDA (Figure 6b). The RDA and pRDA had a similar level of overlap, with 57 top genes in common (6.6%). Top candidate genes from the XtX, pcadapt method or associated to pRDA1 or slope had more chance to be found in putative local ancestry tracts identified in Dallaire et al. (2025), while other local PCA outlier regions were enriched in pcadapt, RDA2, pRDA1, Lake cover, and Sea T° top candidate genes (χ^2^ test, adjusted *p* < 0.05; Table S7).

The higher number of top genes identified by the BF method is linked to the univariate nature of the method, as top‐ranking candidate genes were identified separately based on their correlation with each of the 11 environmental variables. Individual variables had on average 262.1 (SD = 14.7) associated top genes (empirical *p* < 0.001 in the WZA). Out of 2079 unique BF top genes, 571 (27.4%) were associated with more than one variable (404 with two variables, 120 with three, 47 with four or more). Salinity and dissolved O_2_ (55 top genes in common), followed by lake cover and slope (19 genes), were the most frequent pairs.

Across all genome scans and GEA methods, 549 out of 2634 unique top candidate genes (20.8%) were identified by more than one method (Table S8, Figure S9). Principal component analyses based on genetic variation within top candidate genes identified by each method showed highly consistent patterns (Figure S10). These broadly mirrored the genome‐wide structure (Figure 1b), although with reduced resolution and less clearly defined clustering. We tested for enrichment in gene ontology (GO) terms in lists of outlier genes from each test, as well as the list of genes that were outliers according to at least two methods and identified 89 unique GO terms with a *p* value for enrichment under 0.001 and associated with at least three top candidate genes (Table S9). However, after correction for multiple testing, no GO terms presented corrected *p* values under 0.1. 31 unique known QTL positions fell within 100 kb of top candidate genes (Table S8). Those were mostly associated with life history traits, sexual maturation, or head shape in *S. alpinus* , *Oncorhynchus mykiss* , *O. kisutch* , or *S. salar* .

## 4 Discussion

In this study, we aimed to define conservation units for anadromous populations of Arctic Char across its continuous distribution in the Inuvialuit Settlement Region, Nunavut, and Nunavik in Arctic Canada. Through analysis of population structure and connectivity, we highlight two major population groupings, respectively above and below the 67th parallel, which we interpret as having distinct evolutionary trajectories and reduced gene flow. These northern and southern groups were further subdivided into six candidate CUs based on genetic similarity (Figure 2) and connectivity (Figure 4). While we observed interconnected patterns of isolation‐by‐distance and isolation‐by‐environment across the landscape, glacial lineage ancestry was the strongest predictor of genetic distance among populations. Using genome scans and GEA, we detected a polygenic and complex signal of selection under multiple selective pressures, highlighting putatively adaptive variation between the six candidate CUs. A similar hierarchical structure has been reported across the circumpolar range of Arctic Char, including populations in Greenland (Christensen et al. 2018; Madsen et al. 2019) and Russia (Gordeeva et al. 2021), where deep lineage divergence, postglacial recolonization history, and contemporary dispersal jointly shape genomic differentiation. This suggests that the processes structuring diversity in Canadian populations reflect general evolutionary dynamics in the species rather than regional idiosyncrasies.

### 4.1 Hierarchical Population Structure Informs Conservation Units

As a first step toward delimiting conservation units for anadromous Arctic Char, we used a combination of common methods to assess the genetic population structure of our 30 sampling sites. This approach revealed a complex but geographically coherent and hierarchical picture of genetic structure.

At the broadest level, visible in the PCA (PC1) and NGSadmix (*K* = 2) results, the system is defined by an admixture cline from the Arctic lineage in the northwest to the Atlantic lineage in the southeast, as explored in detail in Dallaire et al. (2025). According to mitochondrial data, the divergence time between those lineages could reach up to 400,000 years, predating many glacial cycles (Jacobsen et al. 2022). Regardless of whether admixture occurred during previous interglacial periods, a prolonged phase of allopatry allowed these lineages to diverge significantly, which explains why much of the genetic variation observed in our current dataset reflects this secondary contact. This pattern provides an initial framework for delineating conservation units, as it reveals two distinct evolutionary trajectories: Northern sites are dominated by the Arctic lineage, while Southern sites exhibit varying degrees of admixture between the Arctic and Atlantic lineages. The distinctiveness between these two population segments is also supported by a low effective migration surface between the Kitikmeot and Kivalliq region, as well as Northern and Southern Baffin Island. Although the Northern and Southern populations do not represent entirely pure lineages, we argue that they should be managed as separate broad‐scale CUs. The persistence of this historical signal in contemporary patterns of variation suggests that secondary contact has not erased the imprint of prolonged allopatry. This dominance of ancient divergence over more recent demographic processes is particularly striking in the Arctic, where deglaciation occurred only a few thousand years ago (Dalton et al. 2020), and resembles findings in other post‐glacial populations of fishes such as salmonids (Lehnert et al. 2019) and sticklebacks (Kirch et al. 2021), where deep lineage histories continue to structure modern diversity.

However, our analysis revealed additional hierarchical levels of population structure within the North and South groups, where most individuals showed pure ancestry to a genetic cluster specific to its sampling site. While the Δ*K* summary statistic suggested two groups, studies have shown that this statistic is strongly biased toward *K* = 2 (Janes et al. 2017). In our study, this is not surprising given the overwhelming signal of lineage ancestry that this level of structure represents. Considering that ΔK was stable from *K* = 4–30 and that significant structure between rivers is expected according to the philopatric nature of Arctic Char reproductive migrations (Gyselman 1994; Moore et al. 2013; Dallaire et al. 2021), we argue that most of the 30 clusters identified by NGSadmix could represent populations with some degree of demographic independence. These results highlight the strong hierarchical nature of genetic structure in Arctic Char, as observed in other anadromous salmonids (e.g., Beacham et al. 2004; Habicht et al. 2007; Dionne et al. 2008; Gilbert‐Horvath et al. 2016; Östergren et al. 2021). Importantly, this fine‐scale, river‐specific structure aligns with current fisheries management practices, which often implement river‐specific quotas and management measures to reflect the biological reality of Arctic Char populations.

Our sampling strategy focused on broad coverage across the Canadian range of anadromous Arctic Char rather than fine‐scale resolution. As such, the six candidate CUs proposed here each cover coastal areas ranging from hundreds to thousands of km, which is much larger than current CUs in other anadromous salmonids in southern Canada (e.g., Xuereb et al. 2022; Lehnert et al. 2023). In contrast, Fenton et al. (2026) considered 64 populations of resident or landlocked Arctic Char in the northern British Isles, a study area roughly 14 times smaller than ours. As gene flow is expected to be lower between isolated or non‐migratory populations, they found higher F_ST_ values in neighbouring sampling sites than in the present study. This led to the delineation of 29 ESUs, including multiple ones in single lakes with diverged benthivorous, planktivorous and piscivorous ecotypes. However, any comparison with the current work is hampered by the fact that we limited our assessment of genetic variation to anadromous populations, which have higher value for commercial and subsistence fisheries. Incorporating resident (non‐migratory Chars with access to the sea) and landlocked individuals would likely add more groupings, as gene flow might be reduced between morphs even if they share freshwater habitats (Moore, Loewen, et al. 2014; Salisbury et al. 2018, 2023), but is outside the scope of the present study.

Our analyses uncovered patterns that hold significance for local management of anadromous populations. For example, we identified evidence of straying between most pairs of rivers within 100 km from each other, and in one instance, up to 400 km (one fish sampled at EKA was genetically closer to fish in COP). Despite their philopatric behavior, Arctic Char is known to show less fidelity to natal sites during the upstream migration in non‐reproductive years (Gyselman 1994; Klemetsen et al. 2003; Moore et al. 2013). When migrating strictly for overwintering purposes, fish might select watersheds with easily accessible winter habitats, for example the Ekalluk system in the Kitikmeot Region (EKA, Moore et al. 2017). In the case of AKL and CRB (Kivalliq), fish from either site were indistinguishable using whole‐genome data, which suggests either panmixia between the two systems or that sampling at these sites is not targeting individuals during the upstream reproductive migration, but rather a mixed‐stock of poorly differentiated source populations.

Proper delimitation of MUs in Arctic Char would warrant additional local studies of genetic structure and migration, as was implemented in areas sustaining commercial and subsistence fisheries, such as in Cambridge Bay (Harris, Moore, et al. 2016; Moore et al. 2017), Cumberland Sound (Harris et al. 2014), Paulatuk (Harris, Boguski, et al. 2016), and Ulukhaktok (Lea et al. 2023). Going forward, whole‐genome sequencing is likely not the most cost‐effective method to tackle such local‐scale questions. Alternatively, SNP panels can be developed to assign fish caught in mixed‐stock fisheries to their population of origin, which holds promise for the accurate monitoring of fish stocks and the conciliation of commercial and subsistence fishing (Beemelmanns et al. 2025).

We chose *K* = 7 as an intermediate level of structure that could be used to delimit candidate CUs in Canadian anadromous Arctic Char. This level of structure subdivides our top‐level Northern and Southern CUs, described earlier, and creates genetic groupings that conveniently, but somewhat unexpectedly, closely match the administrative regions of the Canadian Arctic (Figure 1). A notable exception, however, is the union of both the Kivalliq (western) and Nunavik (eastern) coasts of the Hudson Bay in a single cluster (both coasts display signs of common ancestry until *K* = 17), while the rest of the Nunavik populations, around Ungava Bay, appeared as a distinct unit. Considering that *K* = 6 and *K* = 7 discriminated HRN and KJA (Figure S6), our two westernmost sites, we suggest that these two could be paired to form a sixth CU, until additional sampling is performed in the Inuvialuit Settlement Region.

The choice of a level of genetic structure to define CUs implies subjectivity to some degree (Waples 1995), as genetic differentiation typically occurs along a continuum rather than in clear‐cut divisions. In the case of Arctic Char, many populations at the edge of candidate CUs display admixed ancestry to multiple clusters. However, the borders of the proposed CUs were supported by shifts in the dominant cluster that were concordant with areas of low effective migration in our EEMS analyses, as was for example observed in multiple marine species in Atlantic Canada (Wilcox et al. 2023). In the present study, candidate boundaries included the Boothia Peninsula between the Kitikmeot and the North Baffin CUs, and the Foxe Basin and Hudson Strait between the South Baffin and Hudson Bay/Ungava Bay CUs. While EEMS areas of low effective migration do not necessarily imply physical barriers to gene flow (Petkova et al. 2016), they should coincide with shifts in allele frequency that are relevant for informing conservation actions.

### 4.2 Detecting Local Adaptation Using Whole‐Genome Data

We further used our genomic dataset to detect signals of local adaptation in Arctic Char populations to support the delineation of CUs that also reflect patterns of local adaptation to divergent environments (sensu Funk et al. 2012). By using multiple analyses on millions of genetic markers, we obtained an overwhelmingly polygenic signal at the SNP‐level, with numerous peaks of differentiation or associations with the environment across all linkage groups. To summarize this amount of noise and account for the high rate of false positives in the detection of adaptation, we (1) opted for a Windowed Z Analysis to identify top candidate genes while accounting for linkage, and (2) focused on genes that were top candidates in multiple analyses.

As such, we were interested in the relationship between signals of local adaptation and known genomic regions of low recombination, as these might either result from hard selective sweeps or genomic features such as inversions or centromeres (Lotterhos 2019). Our previous work inferred the position of many long haplotype blocks (i.e., sequences containing many polymorphisms inherited as a unit), suggesting a very low recombination rate within those segments (Dallaire et al. 2025). The haplotype frequencies of many of those putative haploblocks were heavily correlated with the admixture gradient between the Arctic and Atlantic glacial lineages. This implies that they act as local ancestry tracts and are much more constrained by post‐glacial demography than spatial variation in selection, though the two are not mutually exclusive. In the partial RDA, we included each population's average ancestry proportion as a covariate in the model, so that the analysis tests only associations between genotypes and environment after factoring out the effect of lineage admixture. Surprisingly, both putative local ancestry tracts and other local PCA outliers (identified in Dallaire et al. 2025) were enriched in top candidates from pRDA1, despite the partial RDA specifically controlling for the effect of Arctic‐Atlantic ancestry.

However, RDA2 and pRDA1 identified common top candidates in a region on LG13 identified as a potential inversion by Dallaire et al. (2025) (Figure 6c). This 290 kb region overlaps seven genes, including insulin‐like 5a and a protein regulating mitochondrial gene, hinting at their importance in metabolism or energy production. The alternative karyotype for this putative inversion is present at higher frequencies in both North and South Baffin, which present contrasted habitats compared to the rest of the Canadian Arctic, with fjord‐like topography (watersheds with a higher slope and lower lake cover) and colder sea‐surface temperatures. It is thus possible that this putative inversion could play a role in adaptation to these environments. Chromosomal inversions suppress recombination and are known to promote co‐adaptation of neighbouring genes in the face of gene flow (Dobzhansky and Sturtevant 1938; Kirkpatrick and Barton 2006; Wellenreuther et al. 2019). This has previously been documented in rainbow trout (*Onchorhyncus mykiss*, Pearse et al. 2019), Atlantic Cod ( *Gadus morhua* , Matschiner et al. 2022), Atlantic Herring ( *Clupea harengus* , Jamsandekar et al. 2024), and Atlantic Silverside ( *Menidia menidia* , Tigano et al. 2021). Although another larger (1.2 Mb) candidate inversion on LG12 is known to be polymorphic in Arctic Char populations from the Kitikmeot (Hale et al. 2021), we did not identify well‐supported top candidate genes within this genomic region.

Another noteworthy genomic region is a ~300 Mb putative haploblock identified in Dallaire et al. (2025) that contains five top candidate genes enriched for SNPs associated with slope or river discharge. Notably, this region overlaps with one highlighted by Salisbury et al. (2023), where the authors reported SNPs with extreme allele‐frequency differences between anadromous and landlocked Arctic Char populations in northern Labrador. In that study, myomesin‐2, lengsin, and calpain‐9 were identified as candidates; in our data, these genes occur in close linkage with additional loci involved in muscle mechanics (e.g., actin alpha skeletal muscle 2, AHNAK‐like, collagen IX) and energy metabolism (e.g., hexokinase‐1, MRPL4, RhoU‐like). The recurrence of this genomic region in a relatively independent geographic dataset (despite evidence for gene flow between systems, Salisbury et al. 2019; Dallaire et al. 2021), and its detection here, without including non‐anadromous populations, further supports its potential importance in adaptation to the physiological demands associated with annual migrations.

When using multiple genome scans and GEA methods with their respective biases, giving greater weight to markers identified by more than one method can help to reduce false positives (Forester et al. 2018). By doing so, we reduced our list of top candidates from 2635 to 541 genes widely distributed across the genome. Despite low statistical support for GO enrichment after correction for multiple testing, we identified key gene functions related to lipid homeostasis and catabolic processes, which ranked among the best‐supported GO categories across multiple candidate gene sets. Lipid metabolism plays a critical role in the adaptation to Arctic climates, supporting membrane fluidity in cold‐water ectotherms such as fishes (Ernst et al. 2016; Wang et al. 2021) and energy reserves during extreme dietary challenges such as prolonged fasting and hibernation (Olsen et al. 2021). The importance of fatty acid homeostasis in local adaptation was also observed in subarctic Atlantic Salmon populations, where genes regulating lipid metabolism were identified as drivers of adaptation (Lehnert et al. 2023). Moreover, because these metabolic pathways are highly sensitive to thermal and oxidative stress (e.g., in Atlantic Salmon, Beemelmanns et al. 2021), the associated genes may serve as critical biomarkers for evaluating population vulnerability and adaptive capacity in the face of climate change, particularly concerning climate‐change induced temperature increases and hypoxia. In addition, intersections between our candidate loci and previously reported QTL in salmonids highlight several genomic regions where independent lines of evidence converge. Such overlap between GEA signals, genome scan outliers, and known QTL suggests that these regions may harbor variants influencing ecologically relevant traits and therefore represent promising targets for future fine‐scale analyses, functional validation, and comparative studies across populations and species.

In summary, identifying the biological processes and molecular functions involved in polygenic adaptation to multiple selective pressures remains challenging (Pritchard et al. 2010; Le Corre and Kremer 2012; Rees et al. 2020). This is particularly true when using indirect inference of selection in the absence of phenotypic data on species with complex life cycles. Here, genome‐wide, high‐density SNP data provided exceptional resolution for detecting candidate signals of selection, but also required substantial aggregation of information (e.g., through the WZA, as recently used in plants, Battlay et al. 2023; and *Drosophila*, Nunez et al. 2024) to make the results interpretable. Integrating multiple analytical methods, genomic context (e.g., recombination landscapes and putative structural variants), and independent sources of evidence allowed us to prioritize a subset of candidate regions where signals converge. This integrative framework highlights that signals of adaptation are not uniformly distributed across the genome but are often concentrated in linked regions that may reflect the joint effects of selection, recombination suppression, and demographic history. As such, combining genome‐wide scans with structural and functional genomic context can help targeting regions of the genome for future fine‐scale and experimental investigations.

### 4.3 Putatively Adaptive Variation Reinforces Candidate CUs

Despite challenges in detecting specific targets for selection, we used the overall putatively adaptive genomic variation to highlight groups of populations that could show local adaptation to similar habitats, thereby supporting the candidate Conservation Units described earlier. For example, the distribution of allele frequencies in relation to environmental variation, as described by the pRDA (Figure 5c,d), emphasizes one of our main findings—that a distinction based on water bodies in the Ungava and Hudson Bay groups was more significant than one based on landmasses (i.e., Nunavik vs. Kivalliq). These waterbodies mainly differed in coastal sea‐surface temperature and salinity, and watersheds flowing into them are topographically distinct: Nunavik watersheds are generally less steep and have higher lake cover than those in South Baffin, our third recommended Conservation Unit in the South Arctic region.

In the North, environmental and geographical distances between populations were too confounded to support isolation‐by‐environment, but putatively adaptive genetic variation supported the distinction between the Kivalliq and North Baffin CUs, as well as the inclusion of the Abernathy Lake (ABL) population, east of the Boothia Peninsula, in the North Baffin CU. Similar to our results on genome‐wide variation, we observed a weaker shift in allele frequencies associated with the environment between Kitikmeot and Inuvialuit Settlement Region populations, putting into question the distinction between these two candidate CUs. However, following a precautionary approach, we recommend maintaining these population segments as separate CUs, with boundaries that could be refined through additional sampling at the western limit of our study area.

Across our study area, conclusions based on genome‐wide, neutral, and putatively adaptive genetic variation were largely consistent, with the adaptive data reinforcing the neutral patterns rather than providing novel insights. While one of the promises of population genomics was to reveal previously undetected adaptive groups that could be considered for conservation (Funk et al. 2012), there is growing evidence that genome‐wide variation effectively predicts adaptive variation in many systems (Chhina et al. 2024). This was observed in other anadromous salmonids like Atlantic Salmon (Moore, Bourret, et al. 2014; Lehnert et al. 2023) and Coho Salmon (Xuereb et al. 2022), and this observation could be influenced by a few key factors. First, polygenic traits are expected to create weak selection signals (as identified in this study) that do not necessarily differ from neutral population structure (Pritchard et al. 2010). In contrast, large‐effect loci such as GREB1L in steelhead (anadromous *O. mykiss* ) and Chinook Salmon ( *O. tshawytscha* ), linked to migration timing, and vgll3 in Atlantic Salmon ( *Salmo salar* ), associated with age at maturity, underlie key phenotypes that are now explicitly considered in conservation and management (Waples et al. 2022).

Second, isolation‐by‐distance patterns suggest limited dispersal over large geographic scales (Aguillon et al. 2017) and straying is likely more frequent among watersheds sharing environmental characteristics. This could be either because of migratory cues, such as water chemistry that differ among regional river systems (Dittman et al. 1996) or because of selection against maladapted migrants (Peterson et al. 2014) at local scales. There is, in fact, a long‐standing hypothesis that homing evolved in anadromous salmonids as it promotes advantageous local adaptations (Quinn 1993; McDowall 2001; Keefer and Caudill 2014). In contrast to the long marine migrations of other anadromous salmonids to offshore feeding areas, Arctic Char feed in coastal waters close to their natal rivers in summer (Dempson and Kristofferson 1987; Spares et al. 2015; Moore et al. 2016), extending the potential for local adaptation to marine habitats in the species (Dallaire et al. 2021). As both freshwater and marine environments tend to be spatially autocorrelated (Legendre 1993), it is not surprising that a significant part of the putatively adaptive genetic variation would be concordant with the neutral structure.

In this study, we reanalyzed genomic data from Arctic Char across the Canadian Arctic and provided a detailed assessment of population structure and connectivity. Although our use of low‐coverage whole‐genome sequencing limited the application of certain approaches (e.g., permutation‐based assessments of differentiation), this strategy is increasingly used in population genomics and is well suited for inferring population structure, connectivity, and genome‐wide patterns of selection when based on genotype likelihood frameworks (Lou et al. 2021). Nevertheless, we note that uncertainty at the individual genotype level may reduce power for some downstream analyses and warrants cautious interpretation of fine‐scale signals. From this analysis, we identified six candidate Conservation Units within two major groups displaying distinct evolutionary trajectories. This framework offers an important baseline for understanding intraspecific diversity at a national level, which can be further refined as additional data become available. Incorporating environmental, ecological, and life‐history information alongside genomic insights will be essential for improving the resolution of these units and ensuring their alignment with meaningful biological boundaries. Furthermore, knowledge co‐production and Inuit traditional ecological knowledge will be invaluable for the future delineation of Conservation Units and overall governance (Cooke et al. 2021). These knowledge systems provide fine‐scale, multigenerational observations of distinct population traits (e.g., size, taste, color, migratory behavior, and spawning phenology) alongside long‐term changes of the environmental conditions that drive local adaptation.

Ultimately, this refined understanding of Arctic Char population structure can guide resource management strategies, ensuring the protection of locally adapted populations and their associated roles in the ecology of northern freshwater ecosystems, their importance for food security, and their contribution to the development of a sustainable economy in the Canadian Arctic. Future analyses that combine genomic baselines with monitoring programs, harvest data, and indigenous traditional knowledge could improve the practical implementation of CUs. Such an approach would allow managers to better anticipate shifts in population dynamics, adjust harvest regulations proactively, and strengthen adaptive co‐management frameworks with northern Indigenous communities.

## Funding

This work was supported by Genome Canada.

## Ethics Statement

We consulted with the indigenous communities providing the biodiversity resources and hired members of local Inuit Hunters and Trappers Associations to help with biodiversity assessments, including the collection of fin clips from Arctic Char harvested for subsistence. The new data generated here is part of our continued engagement to provide genomic resources to these communities to address priority concerns as part of the FISHES project (*Fostering Indigenous Small‐scale fisheries for Health, Economy and food Security*). The findings of this study will be part of outreach reports that will be shared with all partners involved. Lastly, as described above, all data have been shared with the broader public via appropriate biological databases.

## Conflicts of Interest

The authors declare no conflicts of interest.

## Supporting information


**Figure S1:** Principal component analysis of environmental variation for 12 freshwater and marine variables. Sampling sites are colored according to their geographical region.
**Figure S2:** Principal component analysis for genotype likelihoods at 277,570 independent SNPs. a) Proportion (%) of variance explained by the 20 first axes. Individual loadings for b) PC3‐4, c) PC 5‐6, d) PC 7‐8, and e) PC 9‐10. 95% confidence interval ellipses are drawn around each sampling site and the percentage of variance explained by each axis is noted in parenthesis.
**Figure S3:** Unrooted neighbor‐joining tree built on genome‐wide uncorrected p‐distances between Arctic Char samples.
Monophyletic clades where at least 80% of samples originate from a single site (or a pair of neighbouring sites) are collapsed as triangles and truncated at the minimal length. Dashed lines delimit sampling sites, and arrows indicate the origin of samples in smalle clades. Arrows accompanied by fractions denote clades where a portion of individuals were captured at a different site.
**Figure S4:** Unrooted neighbor‐joining tree built on genome‐wide uncorrected p‐distances (bottom axis) between Arctic Char samples. Tip labels indicate sample ID and are colored by administrative regions.
**Figure S5:** (a) Average best likelihood for 50 runs of NGSadmix with K ranging from 1 to 30, with standard deviation in error bars. (b) Δ*K* estimated from the second‐order rate of change in average likelihood divided by the standard deviation. (c) Proportion of 50 runs in the major (black) and first minor (red) mode, as grouped by CLUMPAK with a 0.85 similarity threshold.
**Figure S6:** Proportion of ancestry by individuals for *K* = 2–30 genetics clusters, CLUMPAK‐averaged over replicate runs in the major mode (out of 50 runs). Individuals in all panels are ordered and aligned following the dominant genetic clusters in their sampling site for *K* = 7.
**Figure S7:** Relation between residual Allele Frequency Difference (i.e., after correcting for ancestry distance) and difference in every environmental variable for (a) all, (b) northern, and (c) southern population pairs. Linear regressions were fitted on scatter plots, and the r statistic from a partial Mantel test with marine distance as a covariable (as represented in color from blue to yellow) are displayed. Partial Mantel tests with *p* values under 0.01 after 999 permutations are in red.
**Figure S8:** Edge‐specific effective migration rates (w) estimated from sampling allele frequencies in FEEMS with a smoothing parameter λ = (a) 6.8, (b) 21.5, and (c) 146.8. Sampling sites were repositioned to the nearest node on a triangular grid (cell spacing ≈ 55 km). (d) Average cross‐validation error for λ ranging from 0.1 to 1000. λ values from local minima used in panel a‐c are noted by letters instead of points.
**Figure S9:** (a) Support for selection scans or gene–environment associations on genes with at least 10 SNPs on 4 arbitrarily chosen linkage groups, in 5 panels regrouping methods: (1) XtX (Baypass) or pcadapt; (2) redundancy analysis axes (RDA); (3) partial RDA axes; and Bayes Factors (BF, Baypass) for (4) freshwater and (5) marine variables. Support at the gene level (empirical *p* values) was derived from combined per‐SNP summary statistics in a windowed *Z* analysis (WZA). Genes with the strong support (*p* < 0.001) for more than one type of test were highlighted with bigger diamonds than the rest (small dots). Shaded blue areas mark the position of putative haplotype blocks identified in Dallaire et al. 2025. See Figure 5 for legend.
**Figure S10:** Principal component analysis for LD‐pruned SNPs in top candidate genes detected by each genome scan and gene–environment association methods. 95% confidence interval ellipses are drawn around each site and the percentage of variance explained by each axis is noted in parentheses.

**Table S1:** Information on sampling sites, sampling years and sampling size.
**Table S2:** Summary of environmental variables used for isolation‐by‐environment and Gene–Environment Associations analyses. Collinearity with other environmental variable and glacial lineage ancestry was estimated using Pearson's *r*. Note that Precipitation was noted included in analyses, as it was strongly correlated to Air T°.
**Table S3:** Values for environmental variable, averaged over the catchment area for freshwater variables (green), and over a coastal zone around the sampled river mouth for marine variables (blue).
**Table S4:** Genomic regions identified as outlier after multidimensional scaling (MDS) on local principal component analyses (PCA) for windows of 100 SNPs in Dallaire et al. (2025). Local PCA outlier regions forming three distinct clusters which frequencies in populations correlate to average glacial lineage ancestry were marked as putative local ancestry tracts.
**Table S5:** Pairwise fixation index (FST, below the diagonal) and allele frequency difference (AFD, above the diagonal) between all 30 populations.
**Table S6:** Mantel's r and associated *p* values estimated over 999 iterations for Mantel and partial Mantel tests for the relation between genetic distance (AFD or FST) and marine (km, geographical distance between sampling sites), environmental (Euclidian distance from a principal component analysis of 11 environmental variables), or ancestry (difference in average ancestry for *K* = 2 in NGSadmix) distance matrices in all or either Northern or Southern populations. Environmental distance was further decomposed in individual variables (difference in value between populations) to test the effect of components on isolation‐by‐environment. Tests with *p* values under 0.01 were highlighted in bold and gray.
**Table S7:** Number of top candidate genes by genome scan and Gene–Environment Association test. The number and proportion of those genes overlapping with putative local ancestry tracts and other local PCA outliers from Dallaire et al. (2025) were calculated, and the proportion was compared to the proportion of all genes with at least 5 SNPs overlapping those regions with *χ* ^2^. Proportions in bold and gray are methods where putative local ancestry tracts and other local PCA outliers are enriched in top candidate genes.
**Table S8:** List of top candidate genes (empirical *p* value < 0.001 in a windowed Z analysis) identified by either genome scan or Gene–Environment Association methods. Genes marked as top candidates by multiple methods are highlighted in gray, and overlapping local PCA outlier regions (Table S3, see Dallaire et al. 2025) are noted.
**Table S9:** Gene ontology (GO) terms enriched in top candidate gene lists from each genome scan or Gene–Environment Association method. GO terms with adjusted *p* values under 0.05 are in bold.

## Data Availability

All *Salvelinus alpinus* raw sequencing data analyzed in this manuscript are available on Short Read Archive as part of project PRJNA1031558. A bioinformatical pipeline presenting the main lcWGS analyses is available at: https://github.com/xav9536/angsd_pipeline.
